# Prenatal diagnosis of ectrodactyly-ectodermal dysplasia clefting syndrome ‒ a case report with literature review

**DOI:** 10.1515/crpm-2021-0076

**Published:** 2022-04-07

**Authors:** Egle Savukyne, Egle Machtejeviene, Kotryna Bajeruniene, Virginija Asmoniene

**Affiliations:** Department of Obstetrics and Gynecology, Lithuanian University of Health Sciences, Medical Academy, Kaunas, Lithuania; Department of Genetics and Molecular Medicine, Lithuanian University of Health Sciences, Medical Academy, Kaunas, Lithuania

**Keywords:** case report, ectrodactyly, prenatal diagnosis, TP63 protein, ultrasonography

## Abstract

**Objectives:**

The ectrodactyly-ectodermal dysplasia clefting (EEC) syndrome is a rare genetic anomaly described as ectrodactyly (hands and feet), ectodermal dysplasia, and facial cleft with an incidence of around 1 in 90,000 in the population. This syndrome belongs to the TP63 gene’s mutation family. Ectrodactyly is described as the absence of the central toes or fingers or parts of these appendages. Ectodermal dysplasia usually includes changes in the skin, teeth, hair, nails, endocrine glands, nasolacrimal ducts, genitourinary system, conductive hearing loss.

**Case presentation:**

This is a unique case of a 40-year-old second gravida, suspected of having a sporadic form of EEC syndrome. Routine transabdominal ultrasound at 14 weeks of gestation revealed malformation of the limbs. The two-dimensional and three-dimensional ultrasound at 16 weeks showed a fetus with ectrodactyly of right hand and foot and cleft palate presence. Diagnostic amniocentesis was performed at 17 weeks of gestation. A molecular genetics test using the Sanger sequencing method from amniotic fluid was performed by scanning TP63 gene sequences and revealed a heterozygous pathogenic variant in TP63. The patient decided on feticide.

**Conclusions:**

The heredity of the syndrome is autosomal dominant with high variable expression. More than 300 clinical cases of this syndrome are described in the literature, including both sexes, but the actual etiology is unknown.

## Introduction

The ectrodactyly-ectodermal dysplasia clefting (EEC) syndrome consists of symptoms that include ectrodactyly and syndactyly, ectodermal dysplasia, and cleft lip or/and palate. There are two types of this syndrome: syndrome of the *TP63* gene’s mutation called EEC3 syndrome and syndrome EEC1 due to changes in the 7th chromosome [1]. The heredity of both types of the syndrome varies from partial to complete penetrance [2, 3]. Using sonography, limb buds can be seen transvaginally at eight weeks of gestation, and at 11–12 weeks joints of upper and lower limbs, fingers and toes are visible. Distal limb congenital malformations can be observed either as isolated anomalies involving hands and feet or in the setting of specific syndromes or chromosomal aberrations. Diagnosis of EEC consists of typical phenotype changes and detection of mutations in the *TP63* gene or chromosomal abnormalities.

Commonly the EEC is one at least of six overlapping syndromes and is determined by the mutations of the *TP63* gene. The EEC inheritance is autosomal dominant. However, *de novo* cases can occur in 70% of *TP63*-related diseases [4]. There are three major phenotypes of *TP63* pathogenic variants: ectodermal dysplasia, orofacial clefting, and split-hand/foot malformation [2]. Heterozygous pathogenic *TP63* variants are associated with five different syndromes with overlapping phenotypic features: ectrodactyly, ectodermal dysplasia, cleft lip/palate syndrome (EEC), ankyloblepharon-ectodermal defects-cleft lip/palate (AEC) syndrome, Rapp–Hodgkin syndrome (RHS), acro-dermo-ungual-lacrimal-tooth (ADULT) syndrome, and limb-mammary syndrome (LMS) also rare *TP63* variants are causative for Orofacial Cleft syndrome (OFC8) and non-syndromic split-hand/foot malformation type 4 syndrome (SHFM4) [2].

This is a case of EEC syndrome diagnosed at the 16th week of pregnancy after ultrasound evaluation showing fetus with classic hand, feet and face malformations and confirmed with molecular genetic testing.

## Case presentation

A 40-year-old gravida 2, para 2 patient was administered to the Hospital of the Lithuanian University of Health Sciences (LUHS) Kaunas Clinics, Outpatient department of Obstetrics and Gynaecology for prenatal ultrasound screening because of suspicions of fetal limb anomalies. The first routine ultrasound was performed at a primary health care centre at 14 weeks of gestation, and transabdominal ultrasound revealed malformation of the limbs. The patient and her partner had no other illnesses and family history of hereditary diseases. 10 years ago, from a different partner, she delivered a female newborn with left kidney hypoplasia.

**Figure 1:**
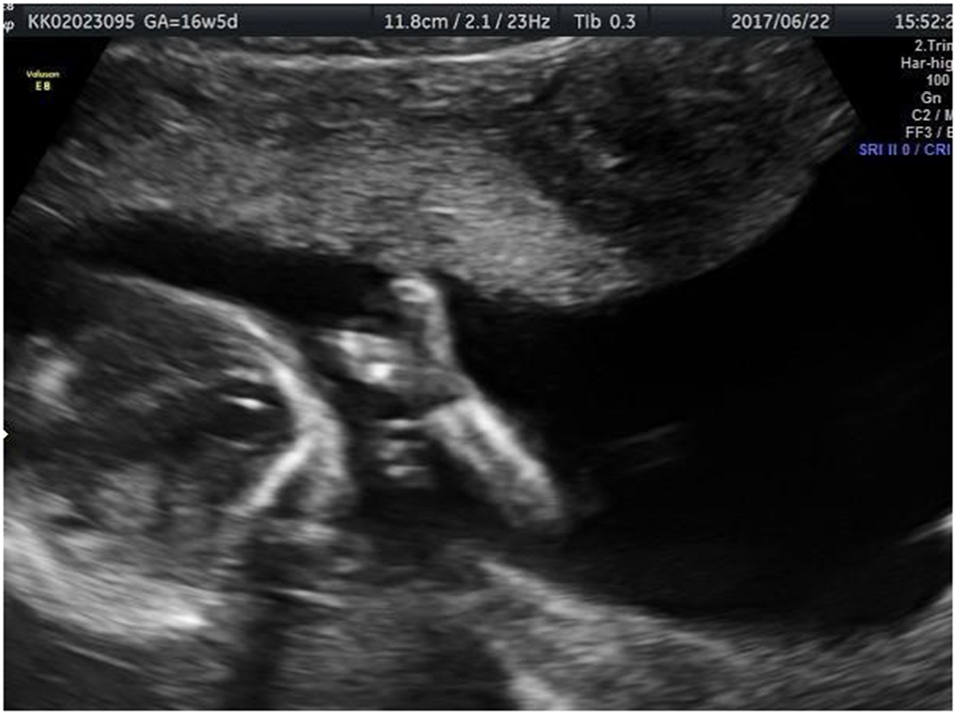
EEC syndrome. Two-dimensional ultrasound demonstrates the lobster-claw hand.

**Figure 2:**
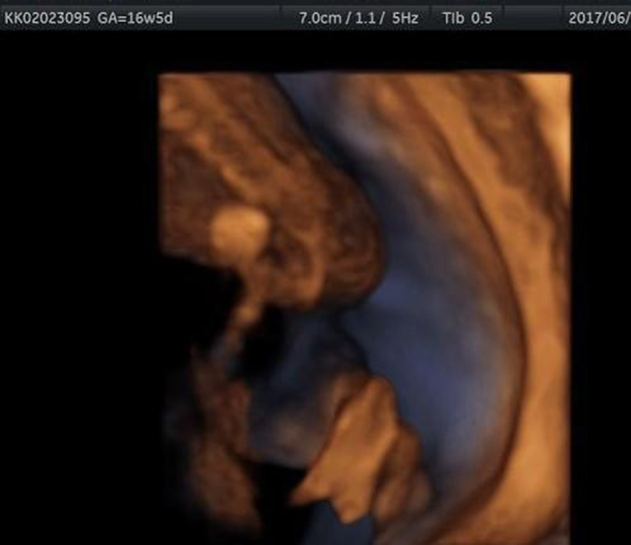
EEC syndrome. Three-dimensional ultrasound in rendering mode demonstrates the lobster-claw foot.

**Figure 3:**
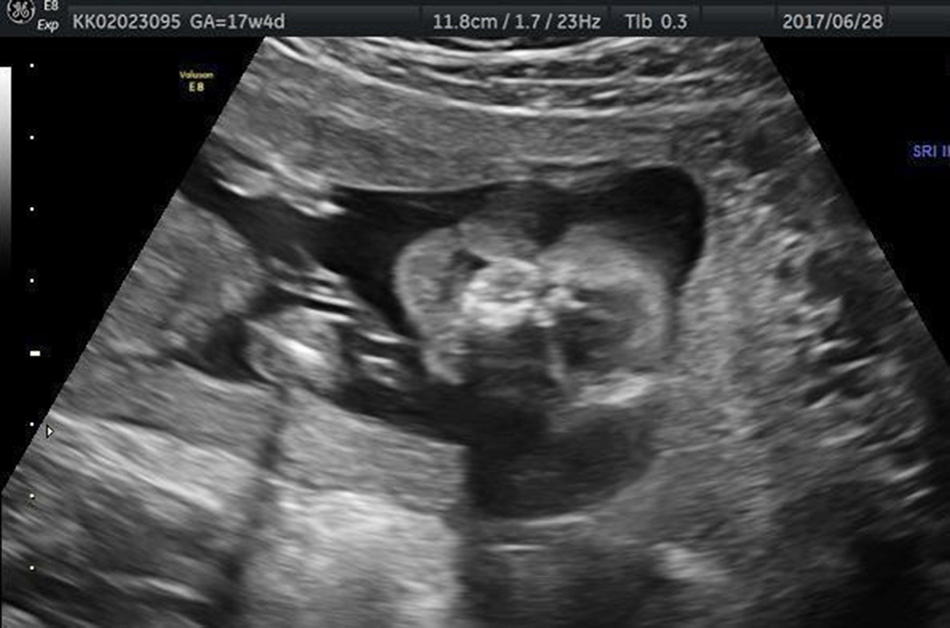
EEC syndrome. Two-dimensional ultrasound demonstrates the cleft lip.

Two and three-dimensional ultrasound was performed at 16 weeks of gestation and ectrodactyly in the right hand (Figure 1) and foot (Figure 2), left kidney pyelectasis. A lip/palate defect (Figure 3) was diagnosed. Biometry results matched gestational age. Diagnostic amniocentesis was performed at 17 weeks of gestation with the resulting karyotype 46, XY.

**Figure 4:**
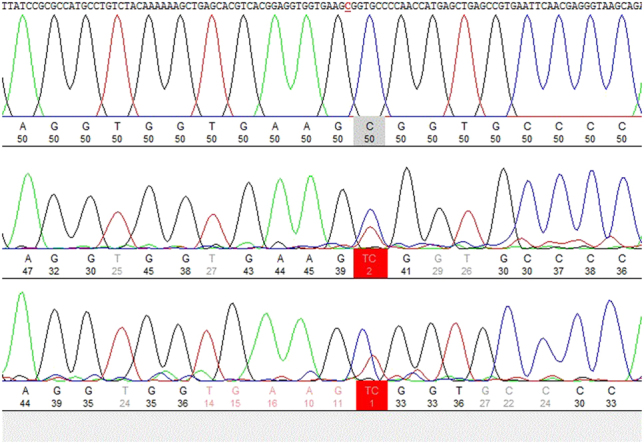
Sanger sequencing electropherogram of *TP63* gene at position 727 shows a pathogenic variant.

A molecular genetics test using the Sanger sequencing method from amniotic fluid was performed by scanning *TP63* gene sequences (*TP63*: E05, E06, E07, E08, E13, E14). According to the manufacturer’s instruction, genomic DNA was isolated from amniotic fluids using a QIAmp DNA blood mini kit. DNA amplification was performed using AmpliTaq Gold 360 PCR mix (6.25 μL), forward and reversed primers for *TP63* exons E05, E06, E07, E08, E13, E14 (1 μL), genomic DNA (1 μL), and deionised water (4.25 μL). Primers were designed using *Primer3* software. The PCR conditions were: initial denaturation step at 95 °C for 10 min followed by 15 cycles (denaturation at 95 °C for 30 s, annealing at 55 °C for 30 s, elongation at 72 °C for 1 min) and final elongation at 72 °C for 10 min. For Dye-terminator sequencing, *BigDye Terminator v3.1 mix* and universal sequencing primers M13 were used. Product separation was performed with Applied Biosystems 3500 Genetic Analyzer; the results were obtained and analysed using *SeqPatient* and *NovoSNP* software against the reference sequence. The sequence analysis revealed a heterozygous pathogenic variant in *TP63* (NM_003722.4): c.727C>T (p.Arg243Trp), which is causative for EEC3 syndrome (Figure 4). This gene encodes transcription factors of p63 and is essential in cell proliferation, differentiation, apoptosis. p63 protein is crucial in early ectodermic tissue development [5].

**Figure 5:**
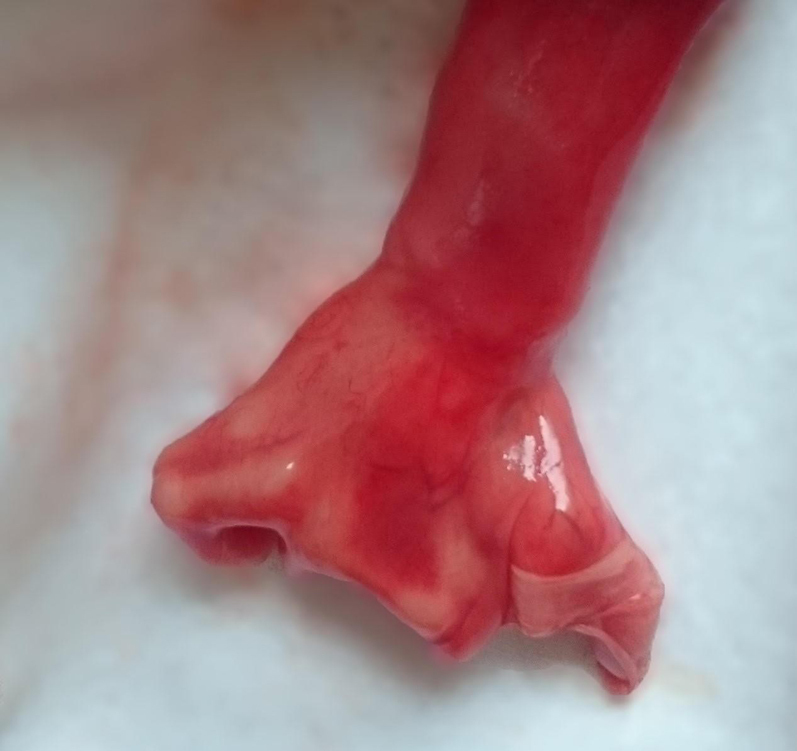
EEC syndrome. Stillborn right-hand demonstrates cleft hand.

**Figure 6:**
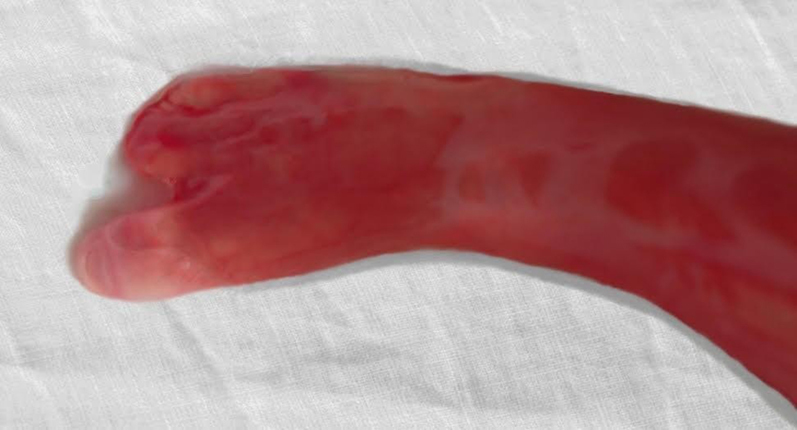
Stillborn foot demonstrates fusion with the first and second toe.

**Figure 7:**
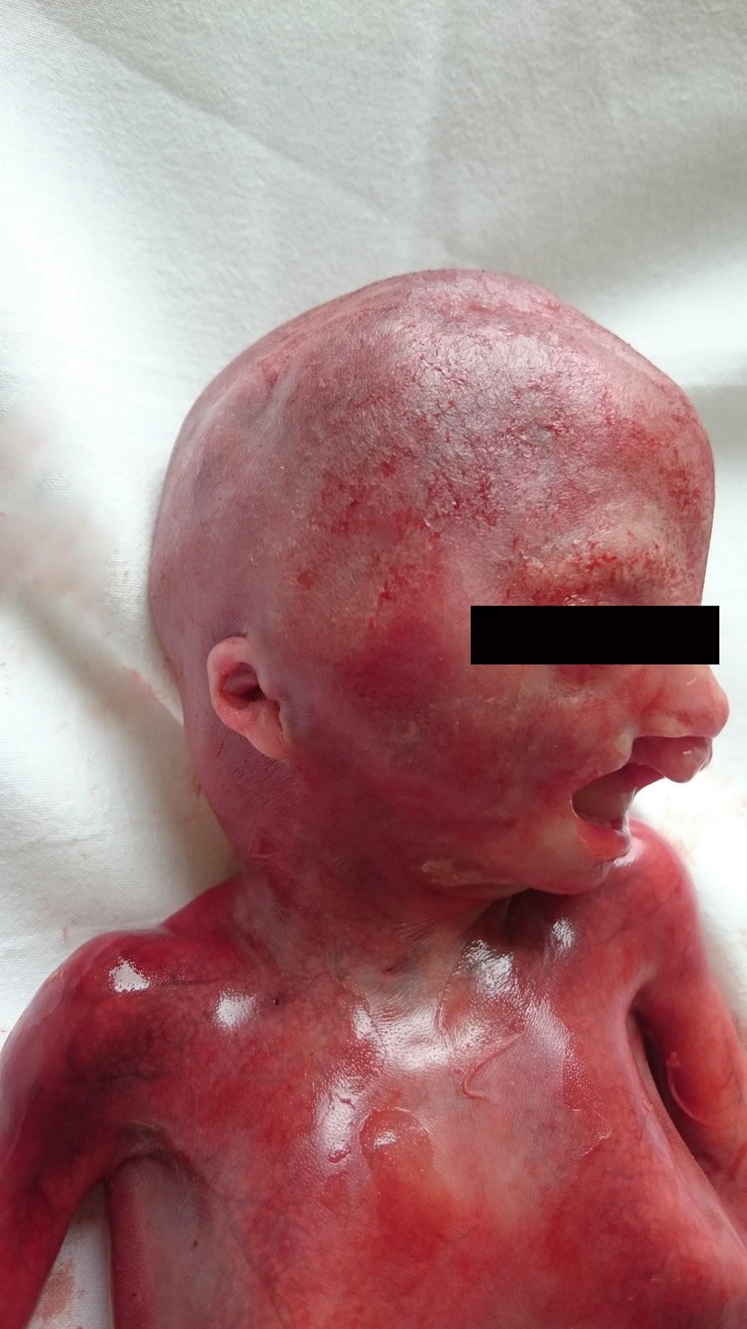
Stillborn face demonstrates cleft lip and palate.

The patient decided on feticide at 22 weeks of gestation. Stillbirth was induced with misoprostol. The patient delivered a male stillborn with multiple dysplasias: absence of the second, third, and fourth fingers on the right hand (Figure 5), presence of the fusions between the first and the second, the third and the fourth toe on the right foot (Figure 6), cleft lip and palate (Figure 7). No other abnormalities were observed, but continued examination was made difficult because of the prematurity of the fetus. No further test was performed at the request of the parents.

## Discussion

Ectrodactyly, ectodermal dysplasia, and cleft lip/palate syndrome is known by various names, including a split hand-split foot–ectodermal dysplasia–cleft syndrome or split hand, cleft hand, or lobster claw hand/foot. This dysplasia contains more than 170 syndromes [1]. In 1970 Rudiger et al. [6] were the first to describe the ectrodactyly-ectodermal dysplasia clefting syndrome. Thurman was the first scientist who described a clinical case of this dysplasia in 1848 [7], but the term ‘ectodermal dysplasia’ was first introduced in 1929 [8].

Ectrodactyly, with or without syndactyly, usually affects central toes or fingers [2]. Face dysplasias, for example, maxillary hypoplasia, can manifest in nose abnormalities, such as broad nasal tip, choanal atresia, the abnormally long groove between the nose and the upper lip. The outer part of the ears can be malformed, resulting in tiny ears [9]. Ectodermal dysplasia affects exocrine glands (sweat, sebaceous, salivary glands), skin, teeth (hypodontia) and hair. Abnormal activity of the salivary glands causes mouth dryness. Lacrimal duct abnormalities can cause increased tearing and vision impairment due to increased risk for eye infections, such as conjunctivitis. Other effects on the eyes can manifest by photophobia, blepharitis, keratitis, corneal ulcerations. Di Iorio et all describes limbal stem cell deficiency (LSCD) as the major cause of ocular morbidity. P63 gene plays a key role in corneal epithelia and explains why mutations might lead to defective limbal stem cell function and progressive keratopathy [10].

Due to sweat glands dysfunction and reduced ability to sweat, fever or heat intolerance can occur. Individuals with EEC syndrome often have dry, discolored, itchy skin, dysplastic nails, hyperkeratosis, coarse, thin, slow-growing hair. They could have no or skinny eyebrows and eyelashes [9].

Endocrine abnormalities can also occur. Some individuals have underdeveloped thymus and hypopituitarism. Glandular abnormalities can result in somatotropin deficiency. The intellectual development of children with EEC syndrome is usually unhindered [9]. Affected individuals may have genitourinary anomalies. Symptoms can include renal agenesis, urethral atresia, and obstruction of the ureters resulting in hydronephrosis. The thin lining of the bladder epithelium that is atrophic or dysplastic can cause dysuria [1].

The EEC inheritance is autosomal dominant and is determined by the mutations of the *TP63* gene. *TP63* gene is localised in the long arm of the 3rd chromosome (3q27–q29). This gene encodes proteins necessary for limbs and ectoderm development. *TP63* gene spans 267 kb, contains 16 exons, encodes a large number of p63 isoforms. But pathogenic variants are found on two *TP63* – the TAp63α and the ΔNp63α isoform (which is shorter and has an alternate N-terminal TA domain (TA^ΔN^)). The full-length *TP63* gene consists of transactivation (TA), DNA binding (DBD), oligomerisation (OD), C-terminal sterile alpha motif (SAM), C-terminal transcription inhibitory (TI) domains [11]. The DNA binding domain is present in all splicing isoforms of p63. All EEC-causing pathogenic *TP63* variants are almost exclusively found in this domain, resulting in abolishing the DNA binding fully or partially in all p63 isoforms that affect the protein’s TA capacity. In contrast, AEC-causing pathogenic variants are found in the SAM or TI, or TA^ΔN^ domains [12].

*TP63* gene has been found to have around six isoforms, with their prime function being modulating gene expression [2]. As it was mentioned above, there are five syndromes reported in the literature caused by mutations in the *TP63* gene and have overlapping phenotypic features. For example, split-hand/foot malformation/syndactyly is the common feature for all *TP63* related syndromes except orofacial cleft eight syndromes (OFC8), meanwhile, Cleft lip/palate is not characterised for ADULT and SHEM4 syndromes [5]. Antenatal diagnosis is feasible using molecular genetic testing, and samples are obtained using chorionic villus sampling or amniocentesis, which should be performed if there is suspicion on fetal ultrasound. Afterbirth diagnosis consists of evaluating phenotype, patient history, and a variety of examinations and tests. For example, X-ray for abnormalities of face and limbs, the ophthalmological exam for potential eye complications, kidney ultrasound, skin biopsy.

Molecular genetic testing can also be performed after birth [1]. Molecular analysis, conducted by sequencing coding regions, identifies from 75 to 99% of all pathogenic variants of *TP63* mutations. If a mutation is not found, it is possible to perform a deletion/duplication analysis to test for rare cases. Nearly 90% of all EEC3 syndrome cases account for missense mutation, causing arginine substitution in exons 6, 7, 8 disrupt DNA binding [4]. In rare cases, frameshift mutation is found in exon 13. Moreover, the location of the pathogenic variants, which commonly are as amino acid substitutions, in the *TP63* gene determines the phenotype in overlapping syndromes: AEC syndrome is caused by mutations in exon 13, and 14 (82% in SAM domain and 18% in ΔNp63-specific N-terminal domain) [13], in ADULT syndrome pathogenic *TP63* gene variants are found in the DNA binding domain, also in ΔNp63α (an alternative TA domain), in TAp63α isoform between the TA and DNA binding domains [14], in LMS syndrome pathogenic missense variants are located between the transactivation (TA) domain and the DNA binding domain (p.Gly115, p.Ser129, p.Gly173 residues in TAp63α isoform) or by truncating variants in the SAM domain [2], in ORF8 syndrome pathogenic *TP63* variants (as large deletion) are found in the DNA binding domain [15].

The treatment of the EEC syndrome is very individual, depending on each patient and the type of the dysplasias. The most of the treatment is symptomatic and sometimes palliative. It is focused on surgical correction of facial and limb deformities. Postnatal problems may involve abnormalities of the tear duct, which can cause corneal damage, as well as anomalies of the hair, teeth, skin, and nails. Because of the great variability in clinical expression, the management of cases of EEC syndrome requires multidisciplinary action. A team of various specialists should participate, including pediatrics, surgeons, plastic surgeons, orthopedics, dentists, speech therapists, ophthalmologists, audiologists, dermatologists, geneticists, psychiatrists, and psychologists [1]. Early interdisciplinary therapy could allow the patients for better social integration.

## Conclusions

Accurate diagnosis of skeletal anomalies can be challenging in the first or sometimes even in the second trimester in the absence of relevant family history. Ultrasound features and fetal karyotyping with molecular genetic testing are the keys to correct diagnosis of EEC syndrome. Molecular genetic testing takes time. Its performance as early as possible, preferably in the first trimester, could allow for the appropriate patient counselling in the second trimester. The case demonstrates the importance of ultrasound screening for detecting fetal anomalies prenatally.
